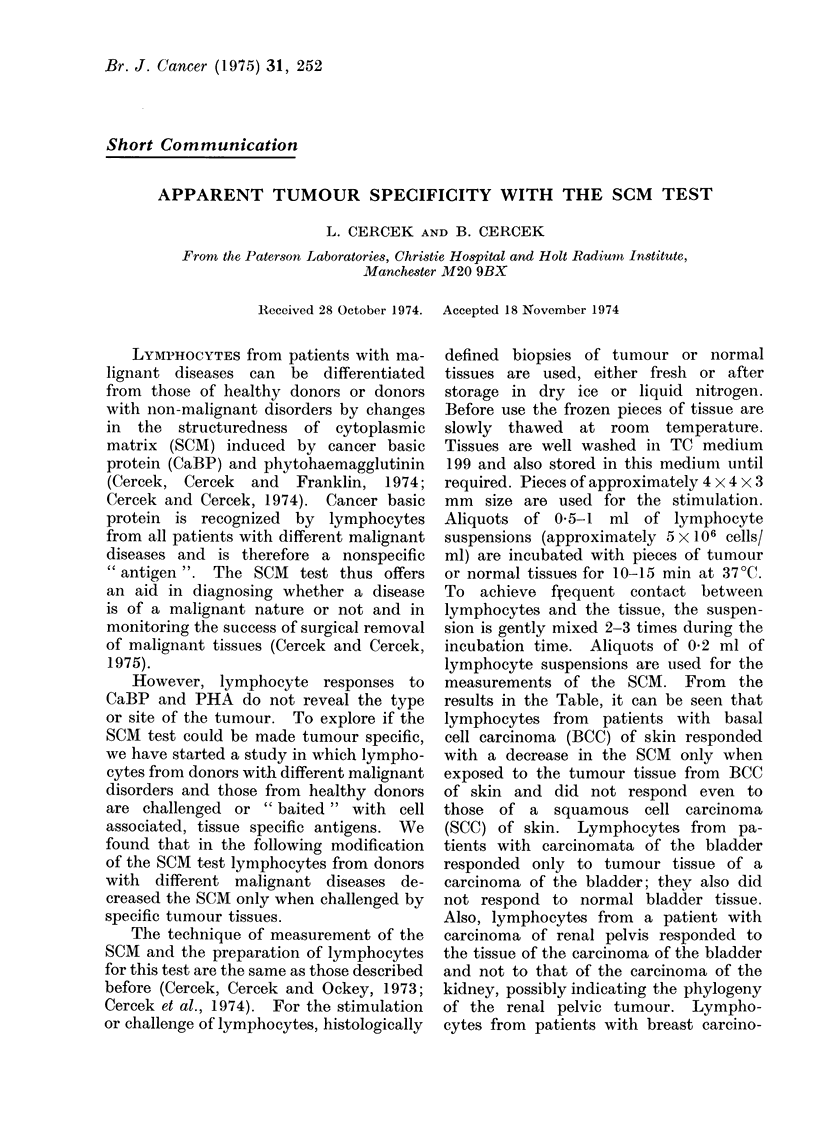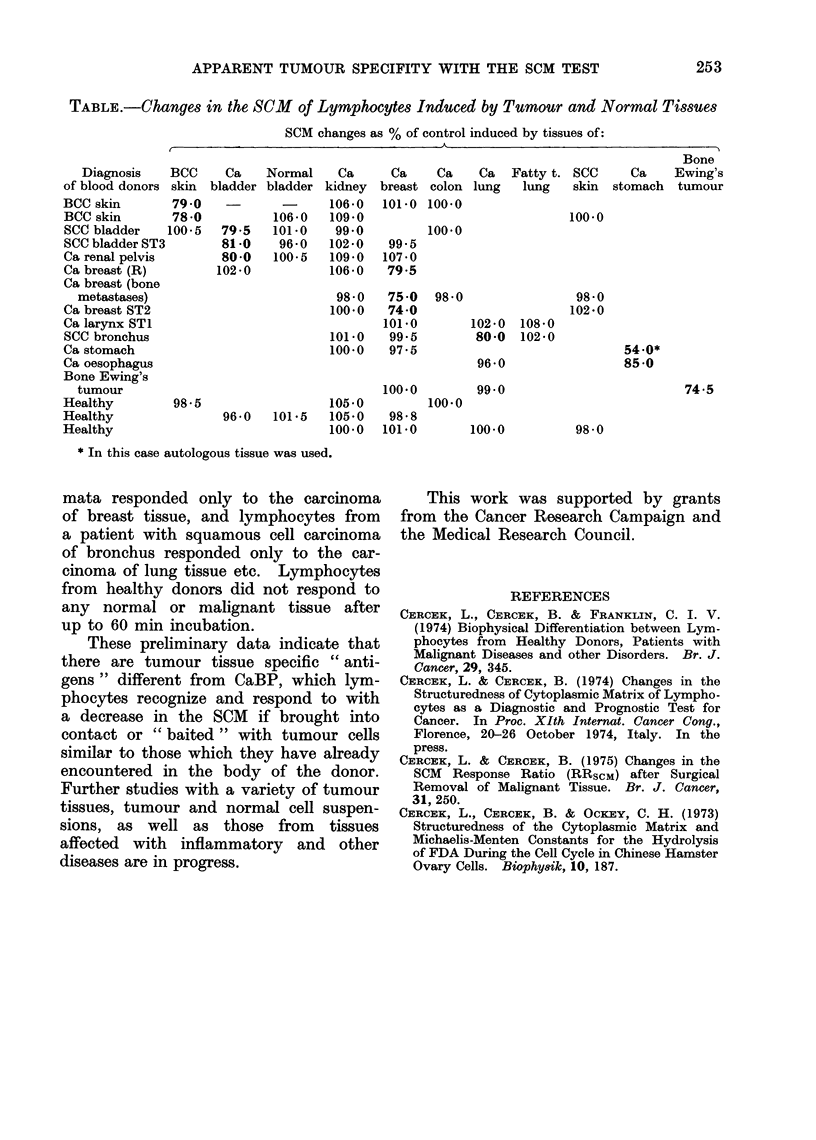# Apparent tumour specificity with the SCM test.

**DOI:** 10.1038/bjc.1975.33

**Published:** 1975-02

**Authors:** L. Cercek, B. Cercek


Br. J. Cancer (1975) 31, 252

***Short Communication***

### APPARENT TUMOUR SPECIFICITY WITH THE SCM TEST

L. CERCEK AND B. CERCEK

*From the Paterson Laboratories, Christie Hospital and Holt Radium Institute, Manchester M20 9BX*

Received 28 October 1974. Accepted 18 November 1974

LYMPHOCYTES from patients with malignant diseases can be differentiated from those of healthy donors or donors with non-malignant disorders by changes in the structuredness of cytoplasmic matrix (SCM) induced by cancer basic protein (CaBP) and phytohaemagglutinin (Cercek, Cercek and Franklin, 1974; Cercek and Cercek, 1974). Cancer basic protein is recognized by lymphocytes from all patients with different malignant diseases and is therefore a nonspecific " antigen ". The SCM test thus offers an aid in diagnosing whether a disease is of a malignant nature or not and in monitoring the success of surgical removal of malignant tissues (Cercek and Cercek, 1975).

However, lymphocyte responses to CaBP and PHA do not reveal the type or site of the tumour. To explore if the SCM test could be made tumour specific, we have started a study in which lymphocytes from donors with different malignant disorders and those from healthy donors are challenged or " baited " with cell associated, tissue specific antigens. We found that in the following modification of the SCM test lymphocytes from donors with different malignant diseases decreased the SCM only when challenged by specific tumour tissues.

The technique of measurement of the SCM and the preparation of lymphocytes for this test are the same as those described before (Cercek, Cercek and Ockey, 1973; Cercek *et al.*, 1974). For the stimulation or challenge of lymphocytes, histologically defined biopsies of tumour or normal tissues are used, either fresh or after storage in dry ice or liquid nitrogen. Before use the frozen pieces of tissue are slowly thawed at room temperature. Tissues are well washed in TC medium 199 and also stored in this medium until required. Pieces of approximately $4 \times 4 \times 3$ mm size are used for the stimulation. Aliquots of 0·5–1 ml of lymphocyte suspensions (approximately $5 \times 10^6$ cells/ml) are incubated with pieces of tumour or normal tissues for 10–15 min at 37°C. To achieve frequent contact between lymphocytes and the tissue, the suspension is gently mixed 2–3 times during the incubation time. Aliquots of 0·2 ml of lymphocyte suspensions are used for the measurements of the SCM. From the results in the Table, it can be seen that lymphocytes from patients with basal cell carcinoma (BCC) of skin responded with a decrease in the SCM only when exposed to the tumour tissue from BCC of skin and did not respond even to those of a squamous cell carcinoma (SCC) of skin. Lymphocytes from patients with carcinomata of the bladder responded only to tumour tissue of a carcinoma of the bladder; they also did not respond to normal bladder tissue. Also, lymphocytes from a patient with carcinoma of renal pelvis responded to the tissue of the carcinoma of the bladder and not to that of the carcinoma of the kidney, possibly indicating the phylogeny of the renal pelvic tumour. Lymphocytes from patients with breast carcino-

TABLE.—*Changes in the SCM of Lymphocytes Induced by Tumour and Normal Tissues*

| Diagnosis of blood donors | SCM changes as % of control induced by tissues of: BCC skin | Ca bladder | Normal bladder | Ca kidney | Ca breast | Ca colon | Ca lung | Fatty t. lung | SCC skin | Ca stomach | Bone Ewing's tumour |
|---|---|---|---|---|---|---|---|---|---|---|---|
| BCC skin | **79·0** | — | — | 106·0 | 101·0 | 100·0 | | | | | |
| BCC skin | **78·0** | | 106·0 | 109·0 | | | | | 100·0 | | |
| SCC bladder | 100·5 | **79·5** | 101·0 | 99·0 | | 100·0 | | | | | |
| SCC bladder ST3 | | **81·0** | 96·0 | 102·0 | 99·5 | | | | | | |
| Ca renal pelvis | | **80·0** | 100·5 | 109·0 | 107·0 | | | | | | |
| Ca breast (R) | | 102·0 | | 106·0 | **79·5** | | | | | | |
| Ca breast (bone metastases) | | | | 98·0 | **75·0** | 98·0 | | | 98·0 | | |
| Ca breast ST2 | | | | 100·0 | **74·0** | | | | 102·0 | | |
| Ca larynx ST1 | | | | | 101·0 | | 102·0 | 108·0 | | | |
| SCC bronchus | | | | 101·0 | 99·5 | | **80·0** | 102·0 | | | |
| Ca stomach | | | | 100·0 | 97·5 | | | | | **54·0*** | |
| Ca oesophagus | | | | | | | 96·0 | | | **85·0** | |
| Bone Ewing's tumour | | | | | 100·0 | | 99·0 | | | | **74·5** |
| Healthy | 98·5 | | | 105·0 | | 100·0 | | | | | |
| Healthy | | 96·0 | 101·5 | 105·0 | 98·8 | | | | | | |
| Healthy | | | | 100·0 | 101·0 | | 100·0 | | 98·0 | | |

* In this case autologous tissue was used.

mata responded only to the carcinoma of breast tissue, and lymphocytes from a patient with squamous cell carcinoma of bronchus responded only to the carcinoma of lung tissue etc. Lymphocytes from healthy donors did not respond to any normal or malignant tissue after up to 60 min incubation.

These preliminary data indicate that there are tumour tissue specific " antigens " different from CaBP, which lymphocytes recognize and respond to with a decrease in the SCM if brought into contact or " baited " with tumour cells similar to those which they have already encountered in the body of the donor. Further studies with a variety of tumour tissues, tumour and normal cell suspensions, as well as those from tissues affected with inflammatory and other diseases are in progress.

This work was supported by grants from the Cancer Research Campaign and the Medical Research Council.

### REFERENCES

CERCEK, L., CERCEK, B. & FRANKLIN, C. I. V. (1974) Biophysical Differentiation between Lymphocytes from Healthy Donors, Patients with Malignant Diseases and other Disorders. *Br. J. Cancer*, **29**, 345.

CERCEK, L. & CERCEK, B. (1974) Changes in the Structuredness of Cytoplasmic Matrix of Lymphocytes as a Diagnostic and Prognostic Test for Cancer. In *Proc. XIth Internat. Cancer Cong.*, Florence, 20–26 October 1974, Italy. In the press.

CERCEK, L. & CERCEK, B. (1975) Changes in the SCM Response Ratio ($RR_{SCM}$) after Surgical Removal of Malignant Tissue. *Br. J. Cancer*, **31**, 250.

CERCEK, L., CERCEK, B. & OCKEY, C. H. (1973) Structuredness of the Cytoplasmic Matrix and Michaelis-Menten Constants for the Hydrolysis of FDA During the Cell Cycle in Chinese Hamster Ovary Cells. *Biophysik*, **10**, 187.